# Examination of the frequency of upward and downward fluctuations in the pressure obtained from the cuff pressure-time curve by continuous measurement of endotracheal tube cuff pressure during thyroid surgery: a case series

**DOI:** 10.1186/s40981-021-00466-4

**Published:** 2021-08-19

**Authors:** Koko Adachi, Yoshinobu Kameyama, Kohkichi Andoh

**Affiliations:** Department of Anesthesiology, Sendai City Hospital, 1-1-1 Asutonagamachi, Taihaku-ku, Sendai, 982-8502 Japan

**Keywords:** Cuff pressure, Thyroid surgery, Postoperative airway complications

## Abstract

**Background:**

Few studies examined time-to-time changes of cuff pressure of an endotracheal tube during surgery. We retrospectively analyzed the changes of cuff pressure during thyroid surgery and examined its relationships with postoperative airway complications.

**Case presentation:**

Cuff pressure was initially adjusted at 26 cmH_2_O and continuously measured in 61 patients. The cuff pressure-time curve dynamically fluctuated, and exceeded 30 cmH_2_O in all patients, whereas decreased to ≤ 20 cmH_2_0 in 42 (69%) patients. Ratio of the period with such an increase and decrease of cuff pressure to the total duration of surgery were 40% (28–66%) and 9% (0–21%), respectively (median, interquartile range). No patients showed symptoms of airway stenosis requiring treatment except one who developed recurrent laryngeal nerve palsy. No patients had lower respiratory tract infection.

**Conclusions:**

Cuff pressure dynamically fluctuated during thyroid surgery. Preventing an increase as well as decrease of cuff pressure is required.

## Background

In recent years, the importance of maintaining optimal endotracheal tube cuff pressure by continuous measurement has been recognized in the field of intensive care [1]. In thyroid surgery, the surgical procedure affects the cuff pressure [2]. Endotracheal tube cuff pressure has been reported to increase, but not decrease, during thyroid surgery [2]. In this study, we showed fluctuations of cuff pressure using the cuff pressure-time curve. We also investigated the frequency of deviation from the optimal cuff pressure and the resulting postoperative airway complications.

## Case presentation

### Methods

This study was approved by the Ethics Committee of Sendai City Hospital (No. 608). We retrospectively examined cuff pressure which was continuously measured during thyroid surgery at Sendai City Hospital from 2016 to 2019. General anesthesia was induced with propofol 1–2 mg/kg, remifentanil 0.2–0.5 μg/kg/min, and rocuronium 0.6–0.8 mg/kg in all cases. After insertion of a spiral tracheal tube (internal diameter 7.0 mm for female and 8.0 mm for male patients), general anesthesia was maintained with propofol 4–7 mg/kg/h, remifentanil 0.2–0.5 μg/kg/min, and a bolus of fentanyl 2–8 μg/kg. After adjusting cuff pressure to 26 cmH_2_O with a pressure gauge following tracheal intubation, it was continuously monitored using a blood pressure monitoring kit connected to the pilot balloon of a tracheal tube. Tracheal tube was removed after administration of sugammadex 200 mg at the discretion of the anesthesiologist on completion of surgery. Cuff pressure-time curve was made based on the recorded data every minute. The duration of cuff pressure ≥ 30 cmH_2_O or ≤ 20 cmH_2_O was measured and expressed as the ratio to the total duration of surgery.

Postoperative airway complications included airway stenosis symptoms and airway infection [1]. Airway stenosis symptoms were defined as subjective findings, such as difficulty in breathing, and objective findings, such as stenotic sounds and tracheal edema confirmed using bronchoscopy. Respiratory tract infections were defined as lower respiratory tract infections requiring postoperative treatment.

Numerical values are presented as median (25% and 75% interquartile range). Statistical analysis was performed by Wilcoxon's sum test using JMP® Pro 16 (SAS Institute Inc., Cary NC, USA) and *P* < 0.05 was regarded as significant.

### Results

**Fig. 1:**
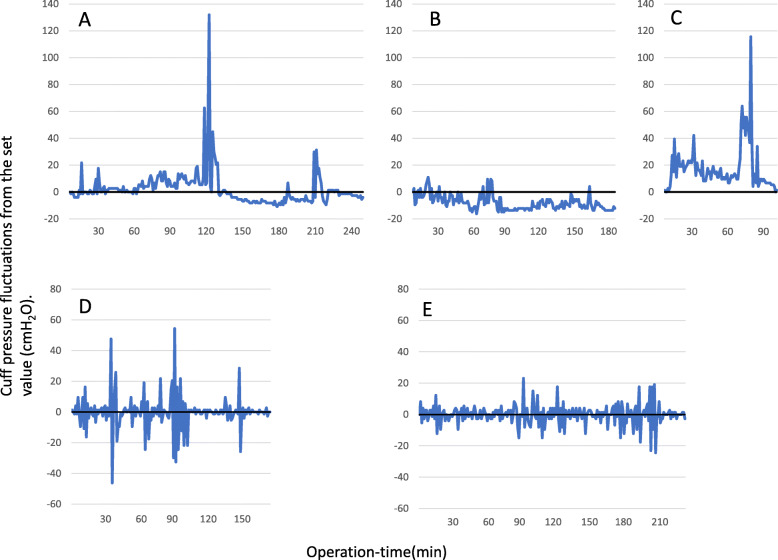
The cuff pressure-time curve. **A**, **D**, and **B** show the cuff pressure-time curve of patients with the maximum, median, and minimum number of times the cuff pressure became 30 cmH_2_O or higher, respectively. **B**, **E**, and **C** show the cuff pressure-time curve of patients with the maximum, median, and minimum number of times the cuff pressure became 20 cmH_2_O or lower, respectively

Of a total of 884 cases, cuff pressure was measured in 61 (16 male and 45 female) cases aged 48 (37–62) years undergoing thyroid surgery for cancer, Graves’ disease, and adenoma (*n* = 31, 19, and 11, respectively). The cuff pressure fluctuated during surgery (Fig. 1). It increased ≥ 30 cm H_2_O in all cases and decreased ≤ 20 cmH_2_O in 42 (69%) patients. Duration of such an increase and decrease of cuff pressure was 40% (28-66%) and 9% (0–32%) of the operation time, respectively. There were five cases of postoperative airway stenosis symptoms, four of which showed difficulty in breathing without findings of airway obstruction. Upon follow-up, the symptoms disappeared. In one case of stenotic sounds, bilateral recurrent laryngeal nerve palsy was confirmed by flexible fiber-optic laryngoscopy after reintubation and extubation. Table 1 shows the relationship between the presence or absence of postoperative airway stenosis symptoms and cuff pressure. There were no cases of lower respiratory tract infection requiring treatment.

**Table 1:** The relationship between the presence or absence of post-operative airway stenosis symptoms and cuff pressure

	The number of times the cuff pressure became 30 cmH_2_O or higher (times)	The ratio to operation time the cuff pressure became 30 cmH_2_O or higher (%)
With airway stenosis symptoms (*n* = 5)	55 (32–92)	40 (16–80)
Without airway stenosis symptoms (*n* = 56)	48 (29–68)	40 (28–66)
*P* value	0.7523	0.9057

### Discussion

In thyroid surgery, cuff pressure is affected by the surgical procedure [2]. There are reports of increased cuff pressure during thyroid surgery [2]; however, to our best knowledge, there is no report stating that the cuff pressure fluctuates upward and downward like a sawtooth wave. This shows that cuff pressure adjustment is necessary to maintain a constant cuff pressure. The use of an automatic cuff pressure regulator that responds to sudden cuff pressure fluctuations can be considered [3].

A cuff pressure of 30 cmH_2_O or higher that may block blood flow in the tracheal mucosa [1, 4] was observed in all cases. The optimal cuff pressure exceeded approximately 40% of the operation time. However, in four of the five cases showing postoperative complications, the difficulty of breathing disappeared during follow-up. In one case of stenotic sound, bilateral recurrent laryngeal nerve palsy was noted postoperatively. There was no association between these symptoms and a cuff pressure of ≥ 30 cmH_2_O (Table 1). There is no report on the duration of the increase in cuff pressure to cause tracheal mucosa ischemia. In this study, the cuff pressure fluctuated upward and downward, similar to the sawtooth wave, and the presence of ischemia release time may be beneficial.

Cuff pressure decreased ≤ 20 cmH_2_O in 42 (69%) patients. This is the first report to show that cuff pressure not only increased but decreased, although there were no cases of respiratory tract infections requiring postoperative treatment. It has been reported that continuous cuff pressure adjustment reduces ventilator-associated pneumonia (VAP) in the intensive care unit [1]. In the future, it will be necessary to consider the relationship between maintaining cuff pressure during surgery and VAP.

The limitation of this study, there were few cases of airway stenosis symptoms, and multivariate analysis could not be performed on the factors that caused airway stenosis. The cause of airway stenosis is thought to be not only the obstruction of blood flow in the tracheal mucosa due to increased cuff pressure, but also the original condition of the trachea and surgical invasion. In the future, the relationship with these factors should be examined by increasing the number of cases. Another limitation is that the cuff pressure was recorded every one minute, which was assumed to be unchanged for one minute and actual time course was not known. However, it was useful to show that the cuff pressures frequently upward and downward fluctuations from the optimal cuff pressure.

### Conclusions

Cuff pressure increased ≥ 30 cmH_2_O in all patients and decreased ≤ 20 cmH_2_O in 42 (69%) patients, for 40% (28–66%) and 9% (0–32) of the total duration of thyroid surgery. Prevention of an increase as well as decrease of cuff pressure would be required. Although this study was not related to postoperative complications, it is necessary to consider measures against not only an increase in cuff pressure, but also a decrease in cuff pressure.

## Data Availability

The datasets used and/or analyzed during the current study are available from the corresponding author upon reasonable request.

## Abbreviation

VAP: Ventilator-associated pneumonia
